# Spectroscopic Properties of TmF_3_-Doped CaF_2_ Crystals

**DOI:** 10.3390/ma17204965

**Published:** 2024-10-11

**Authors:** Carla Schornig, Marius Stef, Gabriel Buse, Maria Poienar, Philippe Veber, Daniel Vizman

**Affiliations:** 1 Crystal Growth Laboratory, Faculty of Physics, West University of Timisoara, 4 Bd. Vasile Parvan, 300223 Timisoara, Romania; carla.schornig76@e-uvt.ro (C.S.); philippe.veber@e-uvt.ro (P.V.); 2 Institute of Advanced Environmental Research, ICAM, 4 Str. Oituz, 300086 Timisoara, Romania; gabriel.buse@e-uvt.ro (G.B.); maria.poienar@e-uvt.ro (M.P.); daniel.vizman@e-uvt.ro (D.V.)

**Keywords:** fluoride crystals, thulium, optical spectroscopy, luminescence

## Abstract

In this study, we report the growth and comprehensive spectroscopic analysis of TmF_3_-doped CaF_2_ crystals, grown using the vertical Bridgman method. The optical absorption and photoluminescence properties of both trivalent (Tm^3+^) and divalent (Tm^2+^) thulium ions were investigated. Optical absorption spectra in the UV-VIS-NIR range reveal characteristic transitions of Tm^3+^ ions, as well as weaker absorption bands corresponding to Tm^2+^ ions. The Judd–Ofelt (JO) formalism was applied to determine the intensity parameters Ω_2_, Ω_4_, and Ω_6_, which were used to calculate radiative transition probabilities, branching ratios, and radiative lifetimes for the Tm^3+^ ions. The emission spectra showed concentration-dependent quenching effects, with significant emissions observed for the concentration of 0.1 mol% TmF_3_ under excitation at 260 nm and 353 nm for Tm^3^^+^ ions and at 305 nm for Tm^2+^ ions. A new UV emission associated with divalent Thulium is reported. The results indicate that higher TmF_3_ concentrations lead to increased non-radiative energy transfer, which reduces luminescence efficiency. These findings contribute to the understanding of the optical behavior of Tm-doped fluoride crystals, with implications for their application in laser technologies and radiation dosimetry.

## 1. Introduction

The spectroscopic properties of TmF_3_-doped CaF_2_ crystals have attracted significant interest due to their potential applications in laser technologies and optical materials [1,2,3,4]. Rare-earth (RE) ions dissolved in CaF_2_, excluding Sm, Eu, and Yb, typically exist in the trivalent state. However, it is known that a certain fraction of these RE^3+^ ions can be reduced to the divalent state through various methods: X-ray irradiation [5,6]; gamma irradiation [7], additive coloration [8], or directly in the as-grown crystals [9,10,11,12,13,14].

Tm^3+^-doped CaF_2_ crystals exhibit efficient near-infrared (NIR) emission, making them suitable for various laser applications [15,16,17,18,19]. Incorporating Tm^3+^ ions into fluoride hosts enhances their spectroscopic properties due to the low phonon energy environment, which reduces non-radiative losses. This makes Tm^3+^-doped CaF_2_ a promising material for developing efficient laser sources.

On the other hand, Tm^2+^-doped CaF_2_ crystals have been investigated especially for the red and near-infrared emission [5,8,20,21].

Previous studies have demonstrated that Tm^3+^-doped CaF_2_ crystals exhibit significant spectroscopic characteristics, including a broad emission band around 1.8 μm under excitation at 795 nm, which are essential for laser applications [16]. The clustering behavior of Tm^3+^ ions in CaF_2_ crystals has also been studied, revealing that clustering can affect the spectroscopic properties of the material [18,19,22]. The energy transfer mechanisms between Tm^3+^ ions in these crystals are crucial for optimizing their performance in laser applications.

The laser performance of Tm^3+^-doped CaF_2_ crystals has been extensively investigated. Studies have shown that these crystals can achieve high slope efficiencies and output powers when used as laser gain media [16,17,18]. Additionally, the co-doping of CaF_2_ crystal with other rare earth ions, such as La^3+^ [23], Y^3+^ [24] or Lu^3+^ [25], can further enhance the near-infrared luminescence performance by enabling efficient energy transfer processes.

Tm^3+^-doped CaF_2_ crystals also exhibit unique thermoluminescent properties, making them suitable for applications in radiation dosimetry [26]. The thermoluminescent response of these crystals under gamma radiation exposure indicates their potential for use in radiation monitoring and measurement.

Studies on Tm^3+^-doped CaF_2_ crystals also reveal their potential for self-Q-switched lasing, demonstrating their versatility in laser technology applications [4,27].

The optical properties of Tm^3+^-doped CaF_2_ have been further studied in different host materials, showing a significant influence of the host in luminescence characteristics [28,29,30,31]. Fluoride crystals with an ordered structure stand out among various host matrices due to their unique chemical, mechanical, and thermal properties. These crystals exhibit a large band gap, broad transparency spectra extending from the vacuum UV to the far-infrared regions, possess low phonon energies that enhance activator luminescence, and feature extended fluorescence lifetimes, among other advantageous characteristics [32,33,34,35,36,37,38]. In the cubic CaF_2_ crystal structure, the F^−^ ions are located at the corners of the cubes. Alternatively, these cubes are occupied with Ca^2+^ ions. Trivalent RE ions substitute for the Ca^2+^ ions and there is a need for charge compensation to maintain electrical neutrality. It has been shown that such charge compensation is enabled by F^−^ ions taking place in the interstitial positions of the empty cubes. At low dopant concentrations (<0.1 wt%), isolated Tm^3+^ ions can be found in several sites resulting from the possible positions for the compensating F^−^ ions in the rare earth ion vicinity. Strickland and Jones [22] identified three major centers in lightly doped 0.05% Tm^3+^:CaF_2_: one center consists of a trivalent ion without any local charge compensation (O_h_ symmetry) and two centers that involve F^−^ charge compensating ions, located as near neighbors or as next near neighbors, the symmetry of these centers being tetragonal (C_4v_) and trigonal (C_3v_), respectively. At slightly higher rare earth dopant concentrations, adjacent rare earth ions appear forming ion pairs (or dimers) and more complicated clusters such as trimers and tetramers depending on the size, nature, and concentration of the considered RE ion [19]. Such a diversity of sites has important consequences on the spectroscopic characteristics of the material, and the very low concentrated systems (<0.05 wt%) must be distinguished from the higher concentrated ones for which ion clusters may be predominant. Despite the fact that there are numerous examples of research on the spectroscopic properties of Tm^3+^ ions in different host materials, there are only a few, older studies on the spectral properties of Tm^2+^ ions in fluorite crystals [19]. A recent study shows the luminescence properties of the Tm^2+^ ions in fluorite crystals. It is reported that an emission in the 680–800 nm range corresponds to 4*f*-4*f* transitions of Tm^2+^ upon excitation by 450 nm laser radiation [20].

In this study, we investigate the spectroscopic properties of TmF_3_-doped CaF_2_ crystals in the UV-VIS spectral region, with a focus on both Tm^3+^ and Tm^2+^ ions, respectively. To achieve this goal, optical absorption and photoluminescence (PL) measurements were performed for both Tm^3+^ and Tm^2+^ ions, and the Judd–Ofelt (JO) model was used to obtain information on the spectroscopic properties of Tm^3+^:CaF_2_ crystals. We focused on the influence of TmF_3_ concentration on the optical absorption and UV-VIS luminescence behavior. Our investigation builds on previous research and aims to provide a comprehensive understanding of the mechanisms that govern the optical behavior of these doped crystals.

## 2. Materials and Methods

**Figure 1:**
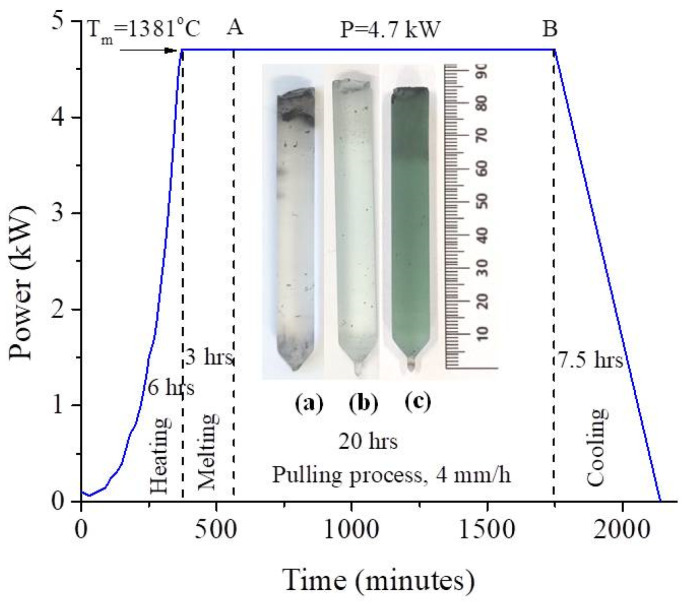
Preparation stage of the Bridgman setup to reach the melting temperature in the graphite heater. The inset shows CaF_2_:x mol% TmF_3_, (**a**) x = 0.1; (**b**) x = 1; (**c**) x = 5.

TmF_3_-doped CaF_2_ crystals were grown in our Crystal Growth Laboratory by the vertical Bridgman method using a shaped graphite furnace which ensures appropriate temperature distribution [39]. The mentioned dopant concentrations refer to the raw material concentration added to the melt. The crystals were grown under a vacuum of approximately 10^−1^ Pa in a graphite crucible. Suprapure grade (Merck) calcium fluoride and thulium fluoride were used as the starting materials. It is known that good charge conversion can be obtained in high deoxidization conditions during the growth process. The use of a graphite crucible during the synthesis can provide a reducing environment that influences the Tm^3^^+^ → Tm^2^^+^ charge conversion in the as-grown samples [40]. Figure 1 shows the typical preparation stages of the Bridgman setup.

The whole process takes place in four stages: (i) the heating stage to reach the melting temperature, T_m_ = 1381 °C (approximately 6 h). The nominal power to reach the melting temperature is P = 4.7 kW. (ii) The melting stage which is necessary to ensure that the entire amount of raw material is melted (approximately 3 h). In stage (iii) the crystal pulling process takes place at a pulling rate of 4 mm/h, at constant power and extends for almost 20 h (AB in Figure 1). The crystal growth begins by lowering the crucible in the temperature distribution in the furnace. The last stage (iv) corresponds to the crystal cooling stage with a cooling rate of approximately (3 ± 0.03) °C/min, which ensures a dislocation density of ~(1 ± 0.1) × 10^4^ dislocations/cm^2^ [41]. The grown crystals are approximately 8 cm long and 10 mm in diameter. The accuracy in measuring the length and diameter of the crystal is 0.01 mm. The crystals were cleaved along the (111) crystallographic direction into several slices. For this study, crystals with thicknesses varying between 1.65 and 2.91 mm were used. They are transparent and free of any visible inclusions or cracks, as shown in the inset of Figure 1a–c. It is observed that with the increase in the concentration of TmF_3_ added in the melt, from 0.1 mol% TmF_3_ (Figure 1a) to 1 mol% TmF_3_ (Figure 1b) and 5 mol% TmF_3_ (Figure 1c), the crystals have an increasingly green color.

The room temperature optical absorption spectra in the UV-VIS-NIR spectral range were recorded using a Shimadzu 1650PC from Shimadzu, Kyoto, Japan and Nexus 470 FTIR spectrophotometer from Thermo Scientific, Cambridge, UK. The baseline correction was applied to the measured absorption spectra in order to eliminate distortions caused by instrument noise, reflectance losses, and stray light. Without this correction, the absorption data would be distorted, leading to overestimated absorption coefficients and inaccurate Judd–Ofelt (JO) analysis. This correction ensured that only the absorption due to the Tm^3^^+^ and Tm^2^^+^ ions was considered, providing a true representation of the material’s optical properties. The raw absorption spectra were first plotted, and baseline points were identified automatically based on the minimal absorption values. A linear fitting method was then applied to define the baseline across the spectrum, ensuring that both broad and narrow features were accounted for. The baseline was subtracted from the original data using the “Subtract” function in OriginLab 9.0, which allowed us to obtain the corrected absorption spectrum accurately representing the TmF_3_-doped CaF_2_ crystals’ true absorption characteristics. To measure the room temperature luminescence spectra, in the UV-VIS domain the PerkinElmer LS55 spectrofluorimeter was used. The luminescence spectra of Tm^3+^ ions were measured by excitation at two wavelengths, 260 and 353 nm respectively, which correspond to the ^3^H_6_ → ^1^D_2_ and ^3^H_6_ → ^3^P_2_ transition [42]. The branching ratios, the emission transition probabilities, and the radiative lifetimes were obtained using the JO analysis (Judd–Ofelt analysis) [43,44]. A comparison with results obtained by other authors was provided. The influence of Tm^3+^ ion concentration on the JO parameters and the radiative lifetime was also investigated. The emission spectra of Tm^2+^ ions were registered under excitation at 305 nm corresponding to the 4f–5d transitions.

## 3. Results

### 3.1. Optical Absorption Spectra

**Figure 2:**
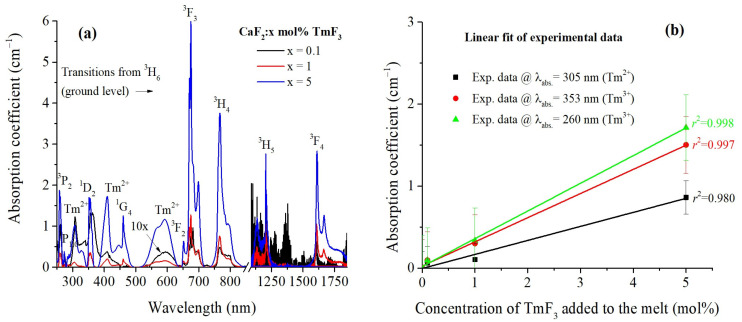
(**a**) Absorption spectra of CaF_2_ doped with three concentrations of TmF_3_ (x = 0.1, 1, and 5 mol%) measured at room temperature. The spectral features correspond to transitions of Tm^3+^ and Tm^2+^ ions, with absorption peaks labelled according to their electronic transitions. The absorption spectrum corresponding to the concentration of 0.1 mol% TmF_3_ is multiplied by 10 (black line); (**b**) Linear fitting of the experimental data. The values of the correlation coefficients, r^2^, are indicated on the graph.

In Figure 2a, the absorption spectra of CaF_2_:x mol% TmF_3_ (where x = 0.1, 1, and 5 mol%) are presented, revealing multiple absorption bands corresponding to both trivalent and divalent thulium ions. To express the absorption spectra in absorption coefficient units, α(λ), the Beer–Lambert law was applied: $\alpha (\lambda )=\frac{2.303\;log_{10}(I/I_{0})}{d}$, where d is the thickness of the sample in cm and $log_{10}(I/I_{0}$) is the absorbance. In order to minimize errors due to optical losses, absorption baseline correction was applied. To be visible, the absorption spectrum corresponding to the concentration of 0.1 mol% TmF_3_ is multiplied by 10 (black line). The observed peaks are attributed to well-known transitions from the ground state, ^3^H_6_, of Tm^3+^ ions (on the basis of Hund’s rules) to the excited states ^3^P_2_ (256 nm) ^3^P_1,0_ (273 nm), ^1^D_2_ (353 nm), ^1^G_4_ (460 nm), ^3^F_2_ (650 nm), ^3^F_3_ (674 nm), ^3^H_4_ (766 nm), ^3^H_5_ (1207 nm) and ^3^H_6_ (1608 nm) of Tm^3+^ ions [42]. For a free Tm^3+^ ion, its electronic configuration is 4f^12^. Along with spectral absorption bands of Tm^3+^ ions in the CaF_2_ host a few weaker absorption bands which correspond to the 4f–5d transition of Tm^2+^ ions are observed, and they are indicated in Figure 2a. These bands are sensitive to the crystal field, which varies with the host lattice (CaF_2_, SrF_2_, BaF_2_), leading to shifts in the energy of the absorption peaks [20]. These maxima are centered around 305, 409, and 593 nm. A linear trend of increasing the absorption coefficient at 353 nm and 260 nm (associated with Tm^3^^+^ ions) and 305 nm (associated with Tm^2^^+^ ions) with the concentration of TmF_3_ was observed. To establish a definitive linear dependence, more measurements across a wider range of concentrations would be necessary. The correlation coefficients are shown in Figure 2b. Also, the error bars were added to each data point, representing the standard deviation from multiple measurements to illustrate the uncertainty in the absorption coefficient values at different TmF_3_ concentrations, thereby providing a clearer depiction of the data’s reliability and supporting the observed linear trends.

### 3.2. Judd–Ofelt Analysis

The standard Judd–Ofelt (JO) analysis was employed for CaF_2_ doped with 1 and 5 mol% TmF_3_ to calculate the spectroscopic parameters based on the optical absorption spectra for Tm^3+^ ions [42,43]. By analyzing six absorption bands corresponding to specific transitions of Tm^3+^ ions from the ground state ^3^H_6_ to excited states: ^3^F_4_, ^3^H_5_, ^3^H_4_, ^2^F_2,3_, ^1^G_4,_ and ^1^D_2_, the JO intensity parameters Ω_2_, Ω_4_ and Ω_6_ were determined. Due to the very low intensity of the absorption bands in the CaF_2_ crystal doped with 0.1 mol% TmF_3_, the JO analysis could not be applied. The parameter Ω_2_ is highly sensitive to the local environment surrounding the doped ions and is related to the asymmetry and covalency of the rare-earth ion sites. The very large difference between the values of Ω_2_ can be explained by the cluster formation with the increase in TmF_3_ concentration. To obtain the JO parameters, Ω_t_ (where t = 2, 4, 6), and the corresponding experimental line strengths (Table 1), a system of six equations was solved using the Levenberg–Marquardt algorithm, based on the six selected transitions. The experimental line strengths were calculated using the following expression:

(1) $$S_{meas}=\frac{3\hbar c\left(2J+1\right)n(\lambda )}{8\pi^{3}N_{0}\lambda_{mean}e^{2}}\left[\frac{9}{{\left({n(\lambda )}^{2}+2\right)}^{2}}\int \alpha \left(\lambda \right)d\lambda \right]$$

where J is the total angular momentum quantum number of the initial state, n(λ) is the refractive index, N_0_ is the Er^3+^ ions concentration (added to the melt), λ_mean_ is the mean wavelength of the specific absorption bands, $\Sigma =\int \alpha \left(\lambda \right)d\lambda$ is the integrated absorption coefficient as a function of λ, α(λ) is absorption coefficient, c is the vacuum speed of light and ħ is Planck’s constant. The refractive index, n, was determined from the Sellmeier dispersion equation [45]. The determination of the JO parameters involved solving a set of six equations for the transitions between the J and J′ manifolds using the experimental line strengths. This calculation is based on the Expression (1) and on the electric dipole (ed) line strength:

(2) $$S_{{JJ}^{\prime }}^{ed}=\sum_{t=2,4,6}\Omega_{t}{\left|\left\langle \left(S,L\right)J\left\|U^{\left(t\right)}\right\|\left(S^{\prime },L^{\prime }\right)J^{\prime }\right\rangle \right|}^{2}$$

and the magnetic dipole (md) line strength given in [29]:

(3) $$S_{{JJ}^{\prime }}^{md}={\left|\left\langle \left(S,L\right)J\left||L+2S|\right|\left(S^{\prime },L^{\prime }\right)J^{\prime }\right\rangle \right|}^{2}$$

where $\left\langle \|U^{\left(t\right)}\|\right\rangle$ are the reduced matrix elements of rank t (t = 2, 4 and 6) of tensor operators between states characterized by the quantum numbers (S, L and J) and (S′, L′ and J′). For selected Tm^3^^+^ transitions, the values of the reduced matrix elements are those tabulated in Kaminskii’s paper [42]. The calculated JO parameters, by applying a least-squares fitting of S_meas_ and S_calc_, and the spectroscopic quality factor, χ, are given in Table 2. A comparison of the obtained JO parameters, Ω_t_, with those reported by other authors [3,23,24,27].

**Figure 3:**
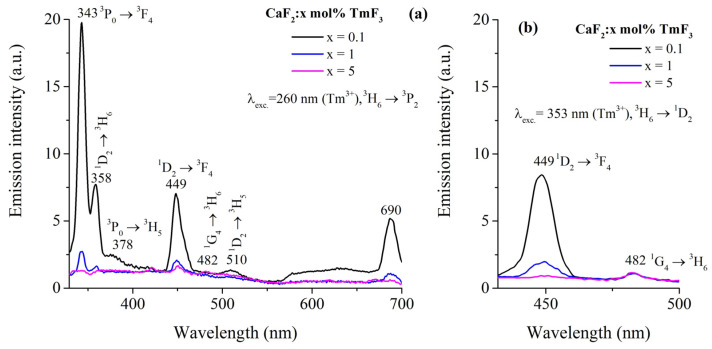
Room temperature emission spectra of CaF_2_ crystals doped with different concentrations of Tm^3^^+^ ions (0.1, 1, and 5 mol%) under excitation at (**a**) 260 nm, corresponding to the ^3^H_6_→^3^P_2_ transition, and (**b**) 353 nm, corresponding to the ^3^H_6_ → ^1^D_2_ transition.

The root mean square deviation, defined by $\Delta S_{\mathrm{rms}}={\left[{\left(q-p\right)}^{-1}\sum {\left(S_{\mathrm{calc}}-S_{\mathrm{meas}}\right)}^{2}\right]}^{1/2}$ is a measure of the accuracy of the fitting procedure; q = 6 is the number of analyzed spectral bands and p = 3 is the number of sought parameters. In this case, ΔS_rms_ = 0.298 × 10^−20^ cm^2^ for CaF_2_:1 mol% TmF_3_ and ΔS_rms_ = 0.267 × 10^−20^ cm^2^ for CaF_2_:5 mol% TmF_3_ which are comparable with those obtained by other authors [3,23,24]. To calculate the radiative lifetime (τ_rad_) for an excited state, J′, the relationship τ_rad_ = 1/ΣA_JJ′_ can be used, where:

(4) $$A_{{JJ}^{\prime }}=\frac{64\pi^{2}e^{2}}{3\hbar (2J+1)\lambda_{mean}^{3}}\left[\frac{n{\left(n^{2}+2\right)}^{2}}{9}S_{{JJ}^{\prime }}^{ed}+n^{2}S_{{JJ}^{\prime }}^{md}\right]$$

is the spontaneous emission probability and the sum is taken over all final lower-lying states, $S_{{JJ}^{\prime }}^{ed}$ and $S_{{JJ}^{\prime }}^{md}$ are defined by Equations (2) and (3). The calculation of fluorescence branching ratios, β_JJ′_ involves utilizing the expression β_JJ′_= A_JJ′·τ_rad__. Table 3 shows the values of the radiative emission probabilities, the branching ratios, and the radiative lifetimes for transitions where luminescence was observed (Figure 3). It should be mentioned that magnetic-dipole transition matters in transitions that obey the selection rules: ΔJ = 0 or ±1, ΔS = 0 and ΔL = 0. Taking absorption spectra into account $S_{{JJ}^{\prime }}^{md}$ is non-zero for ^3^F_2_ → ^3^F_3_, ^3^F_3_→ ^3^F_4_, ^3^H_4_ → ^3^H_5_ and ^3^H_5_ → ^3^F_6_ transitions.

The results obtained using the JO theory suggest that the radiative transition probability of the ^1^D_2_ state is greater than that of other states, as shown in Table 2. This conclusion is in good agreement with Wang et al. [32]. The Judd–Ofelt parameters for different molar contents of TmF_3_, presented in Table 2, provide information on how the local environment of Tm^3^^+^ ions and the symmetry of the crystal field evolve with increasing TmF_3_ concentration. These parameters allow the evaluation of optical transition probabilities and radiative lifetimes of the material, which are directly related to the performance of Tm-doped CaF_2_ crystals in applications such as lasers and phosphors. As the mole fraction of TmF_3_ increases, we can observe changes in the values of Ω_2_, which is sensitive to the asymmetry of the crystal field, indicating how the local structure of Tm^3+^ ions evolves with concentration. The results obtained in this work are compared with those obtained by other authors to provide a basis for understanding how the optical properties of Tm^3+^ in CaF_2_ crystals vary depending on the dopant concentration. [3,23,24,27]. From Table 2, the values of Ω_t_ indicate distinct differences between the samples doped with 1 mol% and 5 mol% TmF_3_. For the 1 mol% TmF_3_ sample, χ = 0.104, which suggests that higher-order transitions Ω_6_ dominate, reflecting a relatively symmetric local environment around the Tm^3+^ ions. In contrast, the 5 mol% TmF_3_ sample shows a significantly higher χ value of 0.857, indicating an increased contribution from moderate angular momentum transitions (Ω_4_), which may be attributed to increased distortion in the local symmetry due to higher dopant concentration. These results suggest that as the concentration of TmF_3_ increases, the local environment becomes more distorted, leading to changes in the relative strength of optical transitions.

### 3.3. Emission Spectra of Tm^3+^ Ions

To obtain the room temperature emission spectra, two absorption bands were pumped, namely λ_exc._ = 260 nm (corresponding to the ^3^H_6_ → ^3^P_2_ transition) and λ_exc._ = 353 nm (^3^H_6_ → ^1^D_2_ transition). The emission spectra of the studied samples under 260 nm excitation are shown in Figure 3a,b. The emission peaks are observed at 343, 358, 378, 449, 482, 510 nm, and 690 nm. These peaks correspond to the following transitions: ^3^P_0_ → ^3^F_4_, ^1^D_2_ → ^3^H_6_, ^3^P_0_ → ^3^H_5_, ^1^D_2_ → ^3^F_4_, ^1^G_4_ → ^3^H_6_, ^1^D_2_ → ^3^H_5_, and ^3^F_2_ → ^3^H_6_. Two emission bands around 449 and 482 nm corresponding to the ^1^D_2_ → ^3^F_4_ and ^1^G_4_ → ^3^H_6_ are observed under excitation at 353 nm.

**Figure 4:**
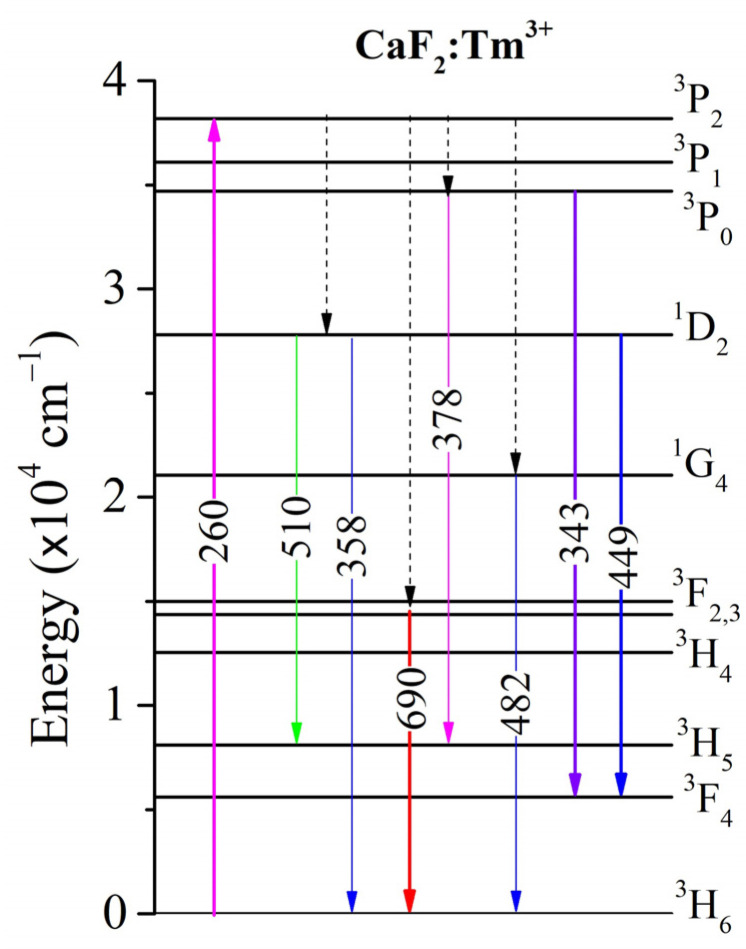
Energy level diagram of Tm^3+^ ions in CaF_2_ crystal illustrating the observed electronic transitions corresponding to the emission peaks in the spectra. The diagram shows excitation processes (solid upward arrows) at 260 nm, leading to the population of the excited states. The subsequent radiative transitions (downward arrows) result in emission at various wavelengths.

The energy level diagram, in Figure 4, provides a comprehensive overview of the electronic transitions that correspond to the emission spectra observed in Figure 3a,b. This diagram allows us to understand the mechanisms behind the different emission peaks and how they relate to the energy levels of the Tm^3+^ ions in the CaF_2_ crystal.

**Figure 5:**
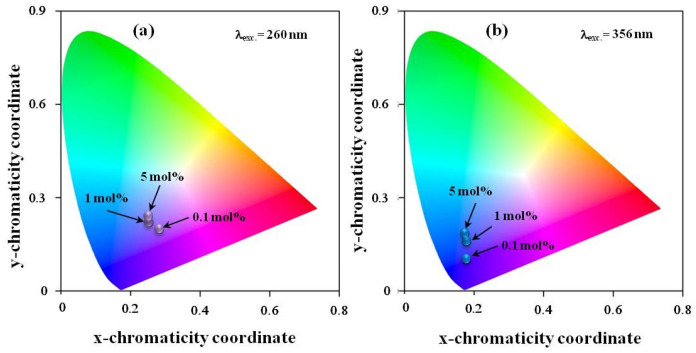
CIE chromaticity diagram of CaF_2_:Tm^3+^ crystal under excitation (**a**) at 260 and (**b**) 356 nm.

The International Commission on Illumination (CIE) chart of the CaF_2_:Tm^3+^ crystal corresponding to the visible emissions is shown in Figure 5a,b for both excitation wavelengths. The color coordinates are shown in Table 4. The results are in good agreement with those reported by Taikar et al. for La_2_O_3_:Tm^3+^ [1]. The calculated color coordinate nearly matched with National Television Standard Committee (NTSC) standard values for blue color. The CIE coordinates were obtained using Gocie V2 software [51].

### 3.4. Emission Spectra of Tm^2+^ Ions

**Figure 6:**
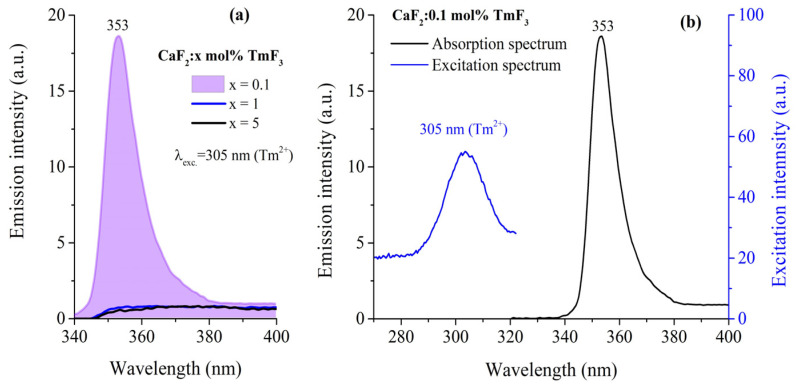
(**a**) Emission spectra of CaF_2_ crystals doped with various concentrations of TmF_3_ (0.1, 1, and 5 mol%) under 305 nm excitation, corresponding to the Tm^2+^ transition. The prominent emission peak at 353 nm is observed, with the intensity significantly decreasing as the concentration of TmF_3_ increases; (**b**) Emission (black-line) and excitation spectrum (blue-line) of CaF_2_: 0.1 mol% TmF_3_.

It is known that the spectroscopic behavior of Tm^2+^ ions is influenced by the host crystal’s structure, the local environment around the ion, and external factors like temperature and irradiation [5,7,20,21,35,37]. Emission spectra of Tm^2+^ ions were investigated in fluorite crystals, especially for the red emission, in the 680–800 nm region [20] under excitation at 450 nm, or infrared emission at 1116 nm [21] and 1250 nm [35]. Figure 6 shows the emission spectra of CaF_2_ crystals doped with various concentrations of TmF_3_ (0.1, 1, and 5 mol%) under 305 nm excitation, corresponding to the Tm^2+^ transitions. The prominent emission peak at 353 nm is observed, with the intensity drastically decreasing as the concentration of TmF_3_ increases.

Figure 6b presents the emission spectrum (black line) and the excitation spectrum (blue line) for CaF_2_ doped with 0.1 mol% TmF_3_. The excitation spectrum, which corresponds to monitoring the emission at 353 nm, shows a clear peak around 305 nm. This suggests that excitation at 305 nm effectively populates the energy levels that lead to the emission observed at 353 nm. The presence of the 305 nm excitation peak is indicative of Tm^2^^+^ ions, as this excitation wavelength aligns with known absorption bands of Tm^2^^+^ rather than Tm^3^^+^. Together, these spectra suggest that the 353 nm emission observed in the figure primarily originates from Tm^2^^+^ ions when excited at 305 nm, supporting the conclusion that the emission at 353 nm is due to transitions of Tm^2^^+^ ions.

## 4. Discussion

The absorption spectrum is a usual method for characterizing the transition properties from the ground state to all excited states of luminescent centers. Additionally, luminescent centers can be identified through their absorption spectra, as each specific center has distinct absorption peaks within a given host material. Figure 2 provides a comprehensive overview of the absorption properties of CaF_2_ crystals doped with various concentrations of TmF_3_, highlighting the distinct absorption behavior of Tm^3+^ and Tm^2+^ ions in the 250–1800 nm spectral region.

The absorption intensity generally increases with the concentration of TmF_3_, particularly evident in the higher concentration samples (x = 5 mol%), which show more pronounced absorption peaks. The spectra demonstrate the successful doping of TmF_3_ into the CaF_2_ matrix, with distinct absorption features consistent with Tm^3+^ and Tm^2+^ ions. The presence of Tm^2+^ bands suggests a partial reduction in Tm^3+^ during crystal growth, which could be of interest for further studies exploring the control of valence states in doped fluoride crystals [18,40]. Figure 2b provides a quantitative analysis of the absorption coefficients at three specific wavelengths: 353 nm (associated with ^3^H_6_ → ^1^D_2_ transition of Tm^3+^ ions), 260 nm (corresponding to the ^3^H_6_ → ^3^P_2_ transition of Tm^3+^ ions), and 305 nm (associated with Tm^2+^ ions). These absorption bands were selected for measuring the emission spectra (see Section 3.3 and Section 3.4). The graph suggests a linear trend between the absorption coefficient and the TmF_3_ concentration, with correlation coefficient values between 0.98 and 0.998. This linear trend can help to estimate the optical properties by adjusting the concentration of TmF_3_ in the host materials. On the other hand, the linearity observed suggests uniform incorporation of Tm ions into the CaF_2_ lattice and minimal clustering, which is essential for maintaining the optical quality of the material. The study of the distribution of trivalent and divalent thulium ions along the CaF_2_ crystals and the determination of the effective segregation coefficients corresponding to both ions in fluorite crystals will be analyzed in a separate paper. The strongest absorption at 674 nm for the highest concentration of TmF_3_ indicates that Tm^3+^ ions dominate the absorption features. On the other hand, the slower increase in the absorption coefficient at 305 nm for Tm^2+^ ions with TmF_3_ concentration suggests a relatively smaller Tm^2+^ ions population increase with TmF_3_ concentration This balance between Tm^3+^ and Tm^2+^ states could be exploited in designing materials for specific photonic applications, such as tunable lasers or optical amplifiers, where controlled absorption is desired.

The emission intensity is highest at the lowest doping concentration (x = 0.1 mol%), particularly for the 343 nm peak, which corresponds to the ^3^P_0_→^3^F_4_ transition. As the doping concentration increases, the emission intensity decreases significantly, indicating a concentration-quenching phenomenon. Taikar et al. [1] reported that after 1 mol% Tm_2_O_3_ was doped in La_2_O_3,_ the emission intensity of Tm^3+^ ions decreases because of concentration quenching. In CaF_2_ crystals, the 1450 nm emission of Tm^3+^ ions is quenched for 1.34% TmF_3_ for which the clusters are predominant. At low doping concentrations (around 0.1 mol%), Tm^3+^ ions are mostly isolated, occupying different lattice sites due to the various possible positions of the compensating F^−^ ions. These isolated Tm^3^^+^ ions, which occupy distinct lattice sites with specific local symmetries, exhibit sharp and well-defined emission spectra because their luminescence is not significantly influenced by neighboring ions. As the concentration of Tm^3+^ ions increases beyond this low doping regime, adjacent Tm^3+^ ions start to form ion pairs (dimers) or more complex clusters (trimers, tetramers, hexamers, etc.). This proximity between Tm^3+^ ions facilitates energy transfer between them, which is one of the primary mechanisms of concentration quenching. This behavior is demonstrated by the experimentally observed fluorescence quenching reported by Renard et al. [19] and Camy et al. [18], which arises directly from these non-radiative processes.

The emission peaks of Tm^3+^ ions observed at 343, 358, 378, 449, 482, 510 nm, and 690 nm, corresponding to the ^3^P_0_ → ^3^F_4_, ^1^D_2_ → ^3^H_6_, ^3^P_0_ → ^3^H_5_, ^1^D_2_ → ^3^F_4_, ^1^G_4_ → ^3^H_6_, ^1^D_2_ → ^3^H_5_, and ^3^F_2_ → ^3^H_6_ transitions were reported previously in La_2_O_3_:Tm^3+^ [32]. To our knowledge, the intense emission band at 343 nm characteristic of the Tm^3+^ ions doped in the CaF_2_ crystal has not been reported before.

By excitation at 353 nm, the emission spectrum is characterized by two emission bands: one of them centered at 449 nm (Figure 3a) which corresponds to the ^1^D_2_ → ^3^F_4_ transition, and the other centered at 482 nm (with a much weaker intensity) corresponding to the ^1^G_4_ → ^3^H_6_ transition of Tm^3+^ ions.

In order to determine manifold-to-manifold radiative emission probabilities, radiative lifetimes and branching ratios the JO theory was applied to the room-temperature absorption spectra. A computer program was designed to perform the least squares fitting between measured and calculated electric dipole line strength of 6 transitions corresponding to the Tm^3+^ ions shown in Table 1. Due to the very low intensity of the absorption bands in the CaF_2_ crystal doped with 0.1 mol% TmF_3_ (see Figure 2), the JO analysis could not be applied. The differences between the theoretical and experimental values can be attributed to the fact that the theoretical values are based on the Judd–Ofelt (JO) theory, which involves certain approximations, such as the assumption of ideal local symmetry around the rare-earth ions (Oh site-symmetry) and homogeneous distribution of dopants within the crystal lattice. In reality, the local environment of Tm^3+^ ions may deviate from these ideal conditions due to lattice defects, clustering, or site distortions, leading to differences between calculated and measured values. At higher concentrations of Tm^3+^ ions, interactions such as cross-relaxation, energy transfer, and ion clustering become significant, affecting the measured transition probabilities. These effects are not typically included in the simplified theoretical calculations, leading to further discrepancies between theoretical and experimental values, especially for transitions like the ^1^G_4_ state. On the other hand, significant errors usually occur in the estimation of JO parameters because it is difficult to obtain accurate absorption line strengths in the case of broad and structured absorption bands (as in our case), and due to the JO model itself [52]. These large errors have also been reported in other papers [24,30] for Tm^3+^ ions.

The JO parameters are listed in Table 2 along with the JO parameters found in previous studies [3,23,24,27]. The results obtained suggest that the radiative transition probability of the ^1^D_2_→^3^F_4_ transition (~2049 s^−1^) is greater than that of other states, as shown in Table 2. This result is in good agreement with the emission spectrum under excitation at 353 m (Figure 3b). However, the strong emission around 343 nm under excitation at 260 nm (^3^H_6_→^3^P_2_) cannot be explained by JO analysis. Further investigations are needed to clarify this.

The UV emission spectra of Tm^2^^+^-doped CaF_2_ crystals under 305 nm excitation (Figure 6) show a pronounced peak at 353 nm for CaF_2_:0.1 mol% TmF_3_. To our knowledge, the UV emission of Tm^2+^ ions has not been reported before. The intensity of this peak decreases significantly with increasing TmF_3_ concentration, from 0.1 mol% to 5 mol%, suggesting the presence of concentration quenching. This quenching effect can be attributed to the increased probability of non-radiative energy transfer between Tm^2+^ and Tm^2+^/Tm^3+^ ions, which becomes more pronounced at higher doping levels. The observed spectral characteristics align with previous studies on Tm^2+^ in CaF_2_, which highlight the role of crystal field splitting and the importance of maintaining optimal doping concentrations to preserve luminescence efficiency [35]. These findings contribute to our understanding of the spectroscopic behavior of Tm^2+^ in fluoride hosts, with implications for their use as a luminescent material.

## 5. Conclusions

In this work, we successfully grew TmF_3_-doped CaF_2_ crystals using the vertical Bridgman method and investigated their spectroscopic properties. The optical absorption spectra revealed distinct transitions of both trivalent (Tm^3+^) and divalent (Tm^2+^) thulium ions, providing insights into the nature of the doped ions and their interactions within the host matrix. The application of the Judd–Ofelt theory enabled us to determine the spectroscopic parameters, including intensity parameters, radiative transition probabilities, branching ratios, and radiative lifetimes which are useful for understanding the luminescence mechanisms in these materials. Our photoluminescence studies demonstrated that TmF_3_-doped CaF_2_ crystals exhibit strong UV-VIS emissions, with significant emission quenching observed at higher dopant concentrations due to increased non-radiative energy transfer. This quenching effect highlights the importance of optimizing dopant concentrations to maximize the luminescence of Tm^3+^ and Tm^2+^ efficiency in CaF_2_ crystals. A new UV emission around 353 nm, associated with divalent Thulium, was reported. To our knowledge, the intense emission band at 343 nm characteristic of the Tm^3+^ ion doped in the CaF_2_ crystal has not been reported before. The observed luminescent properties, particularly the emission peaks associated with both ions, suggest that these materials hold great potential for applications in laser technologies. Overall, our findings provide an understanding of the spectroscopic behavior of TmF_3_-doped CaF_2_ crystals, contributing to the knowledge of the field of rare-earth-doped fluoride materials. Future work will focus on exploring the control of valence states in these crystals and optimizing their optical properties for specific photonic applications.

## Figures and Tables

**Table 1:** The transition, mean wavelength and the integrated absorption cross-section of the measured and calculated absorption line strengths of the selected absorption peaks of CaF_2_:TmF_3_ crystals.

Sample		CaF_2_:1 mol% TmF_3_			CaF_2_:5 mol% TmF_3_		
Transition, ^3^H_6_→	λ_m_ [nm]	Σ [×10^−20^ cm^2^·nm]	*S*_meas_ [×10^−20^ cm^2^]	*S*_calc_ [×10^−20^ cm^2^]	Σ [×10^−20^ cm^2^·nm]	*S*_meas_ [×10^−20^ cm^2^]	*S*_calc_ [×10^−20^ cm^2^]
^3^F_4_	1787	4.027	1.437	1.211	15.605	0.933	0.783
^3^H_5_	1234	14.1428	1.248	1.093	8.125	0.702	0.885
^3^H_4_	785	6.960	0.944	1.098	6.806	0.923	0.978
^2^F_2_ + ^2^F_3_	677	9.089	1.429	1.267	9.146	1.436	1.148
^1^G_4_	460	1.148	0.264	0.087	1.402	0.323	0.089
^1^D_2_	353	1.722	0.513	0.181	1.669	0.498	0.368
			Δ*S*_rms_ = 0.298 × 10^−20^ cm^2^			Δ*S*_rms_ = 0.267 × 10^−20^ cm^2^	

**Table 2:** The Judd–Ofelt parameters, Ω_t_ (expressed in 10^−20^ cm^2^) and the spectroscopic quality factors, χ.

Ω*_t_* (10^−20^ cm^2^)	CaF_2_:1 mol% TmF_3_ (This Paper)	CaF_2_:5 mol% TmF_3_ (This Paper)	CaF_2_: 0.5 at.% TmF_3_ [24]	CaF_2_:1.5 at.% TmF_3_, 4 at.% YF_3_ [3]	CaF_2_:3 at.% TmF_3_, 2 at.% LaF_3_ [23]	CaF_2_:3 at.% TmF_3_, 2 at.% LaF_3_ [27]
Ω_2_	1.25043	0.20294	3.28	0.891	1.429	1.283
Ω_4_	0.15075	0.88504	0.99	1.251	1.485	1.323
Ω_6_	1.44999	1.03299	1.52	0.959	1.163	2.159
χ	0.10397	0.85677	0.65	1.304	1.277	0.590

**Table 3:** The transitions corresponding to the wavelengths where luminescence was observed (Figure 3a,b), along with the calculated radiative lifetimes, radiative emission probabilities, and branching ratios.

Transition	λ_em._ (nm)	Ref.	CaF_2_:1 mol% TmF_3_			CaF_2_:5 mol% TmF_3_		
			τ_calc._ (ms)	A_JJ′_ (s^−1^)	β_JJ′_	τ_calc._ (ms)	A_JJ′_ (s^−1^)	β_JJ′_
^3^F_3_→^3^H_6_	690	[3,32]	0.87	1010	0.87 ± 0.173	0.95	915.4	0.87 ± 0.129
^1^D_2_→^3^H_5_	510	[1,32]	0.20	78.7	0.02 ± 0.003	0.20	58.5	0.01 ± 0.002
^1^G_4_→^3^H_6_	482	[1,3,32,46,47,48,49,50]	0.83	175.7	0.15 ± 0.030	1.23	157.5	0.22 ± 0.033
^1^D_2_→^3^F_4_	449	[1,3,32,46,47,48,49,50,51]	0.20	2048.8	0.41 ± 0.081	0.20	1137.6	0.23 ± 0.034
^3^P_0_→^3^H_5_	378	[32]	0.78	0.10	-	0.95	0.10	-
^1^D_2_→^3^H_6_	358	[3,46]	0.20	1508.8	0.30 ± 0.060	0.20	3074.3	0.63 ± 0.093
^3^P_0_→^3^F_4_	343	[32]	0.78	11.4	0.01 ± 0.002	0.95	66.8	0.06 ± 0.009

**Table 4:** The CIE coordinates chart of the CaF_2_:Tm^3+^ crystal corresponding to the observed visible emissions under excitation at 260 and 356 nm.

CaF_2_:*x* mol% TmF_3_	λ_exc._ (nm)	X-Coordinate	Y-Coordinate	λ_exc._ (nm)	X-Coordinate	Y-Coordinate
*x* = 0.1	260	0.28	0.20	356	0.18	0.10
*x* = 1		0.25	0.22		0.18	0.16
*x* = 5		0.25	0.24		0.17	0.19

## Data Availability

The data presented in this study are available on request from the corresponding author. The data are not publicly available due to privacy reasons.
